# Women’s perspectives on the acceptability and feasibility of an HPV screen-and-treat approach to cervical cancer prevention in Iquitos, Peru: a qualitative study

**DOI:** 10.1186/s12905-022-01943-3

**Published:** 2022-10-10

**Authors:** Rachel M. Morse, Joanna Brown, Helen E. Noble, E. Jennifer Ríos López, Anna Kohler-Smith, Sandra Soto, Daniel Lenin del Cuadro, Karina Gonzales Díaz, Magaly Figueredo Escudero, Giannina Vásquez del Aguila, Lita E. Carrillo Jara, Hermann F. Silva Delgado, Victor A. Palacios, Carlos Santos-Ortiz, Patti E. Gravitt, Valerie A. Paz-Soldan, the Proyecto Precancer Study Group, Meda Del Carpio-Morgan, Esther Y. Garcia Satalay, Sarah D. Gilman, José Jerónimo, Alcedo Jorges, Magdalena Jurczuk, Margaret Kosek, Gabriela Ladrón de Guevarra, Renso Lopez Liñán, Andrea Matos Orbegozo, Jaime Marín, Graciela Meza, Reyles Ríos Reátegui, Karina Román, Anne F. Rositch, Nolberto Tangoa, Javier Vásquez Vásquez, Giannina Vásquez del Aguila, Karen Zevallos

**Affiliations:** 1 Department of Tropical Medicine, Tulane University School of Public Health and Tropical Medicine, New Orleans, LA USA; 2 Asociación Benéfica PRISMA, Lima, Peru; 3 Global Health Fogarty International Program, University of Washington Northern Pacific, Seattle, WA USA; 4 Department of Cancer Control and Prevention, Gerencia Regional de Salud de Loreto, Iquitos, Loreto Peru; 5 Facultad de Medicina Humana, Universidad Nacional de la Amazonía Peruana, Iquitos, Peru; 6 Dirección de Prevención y Control de Cáncer, Ministerio de Salud, Lima, Peru; 7 Department of Epidemiology and Public Health, University of Maryland School of Medicine, Baltimore, MD USA; 8 Laboratorio, Centro de Salud de San Juan, Iquitos, Peru; 9 School of Medicine and Health Sciences, George Washington University, Washington, DC USA; 10 US National Cancer Institute, Bethesda, MD USA; 11 Division of Infectious Diseases and International Health, Department of Internal Medicine, University of Virginia, Charlottesville, VA USA; 12 Hospital Regional de Loreto, Loreto, Peru; 13 Department of Epidemiology, Johns Hopkins Bloomberg School of Public Health, Baltimore, MD USA

**Keywords:** Acceptability, Ablative therapy, Cervical cancer, Screen-and-treat, Thermal ablation

## Abstract

**Background:**

The objective of this study was to explore women’s experiences of a screen-and-treat approach with ablative therapy (referred to by the Spanish acronym TVT-TA) as a method of treatment following a positive HPV test in Iquitos, Peru.

**Methods:**

A total of 111 in-depth interviews were conducted with 47 HPV positive women who attended the TVT-TA procedure at a primary-level healthcare facility. Interviews were conducted immediately before, immediately after, and six-weeks after TVT-TA.

**Results:**

Most interviewed women reported experiencing moderate pain during ablative therapy and minimal pain immediately after and six weeks after ablative therapy. Women also stated that the pain was less intense than they had expected. The most common physical after-effects of treatment were bleeding and vaginal odor. Women experienced oscillating emotions with fear upon receiving a positive HPV result, calming after hearing about ablative therapy treatment, worry about pain from the treatment itself, relaxation with counseling about the procedure, and relief following treatment.

**Conclusions:**

Nearly all participants emphasized that they were pleased with the TVT-TA process even if they had experienced pain during TVT-TA, recommended that TVT-TA be expanded and available to more women, and stated that TVT-TA was faster and easier than expected. This study found that TVT-TA is a feasible and acceptable means of treating HPV according to the women receiving the treatment.

## Background

Cervical cancer is a highly preventable form of cancer that is estimated to lead to the deaths of 338,800 women globally each year [1], disproportionately affecting women in low- and middle-income countries (LMICs) where 91% of these deaths occur [2]. In the Loreto district of Peru, the cervical cancer mortality rate is 2.6 times higher than the global average and contributes to more cancer related deaths among women than any other form of cancer [3–6].

The World Health Organization’s (WHO) goal to eliminate cervical cancer (< 4 per 100,000) by 2030 focuses on three complementary and necessary elements: human papillomavirus (HPV) vaccination of 90% of girls before the age of 15 years, screening for 70% of eligible women (particularly those 35–45 years of age), and appropriate follow up or treatment for 90% of women with abnormal screening results [7]. Unfortunately, data from many LMICs, including Peru, show not only inadequate screening rates but very limited follow up and treatment for screen positive women [8–12].

In 2013, the WHO published guidelines in support of the use of ‘screen-and-treat’ approaches in cervical cancer early detection and treatment (EDT) programs [13]. These guidelines aim to address systems-level challenges preventing women from receiving follow up care or treatment following a positive screening result. One of the WHO suggested approaches was HPV testing for the presence of high-risk HPV, followed by triage screening using visual inspection with acetic acid to determine eligibility for ablative therapy. In this strategy, women who test HPV positive and are eligible are treated with ablative therapy. Ineligible women are referred for additional follow up [13]. The WHO has since released additional evidence-based guidelines providing further support for the use of ablative therapy to treat precancerous cervical lesions [2].

**Fig. 1:**
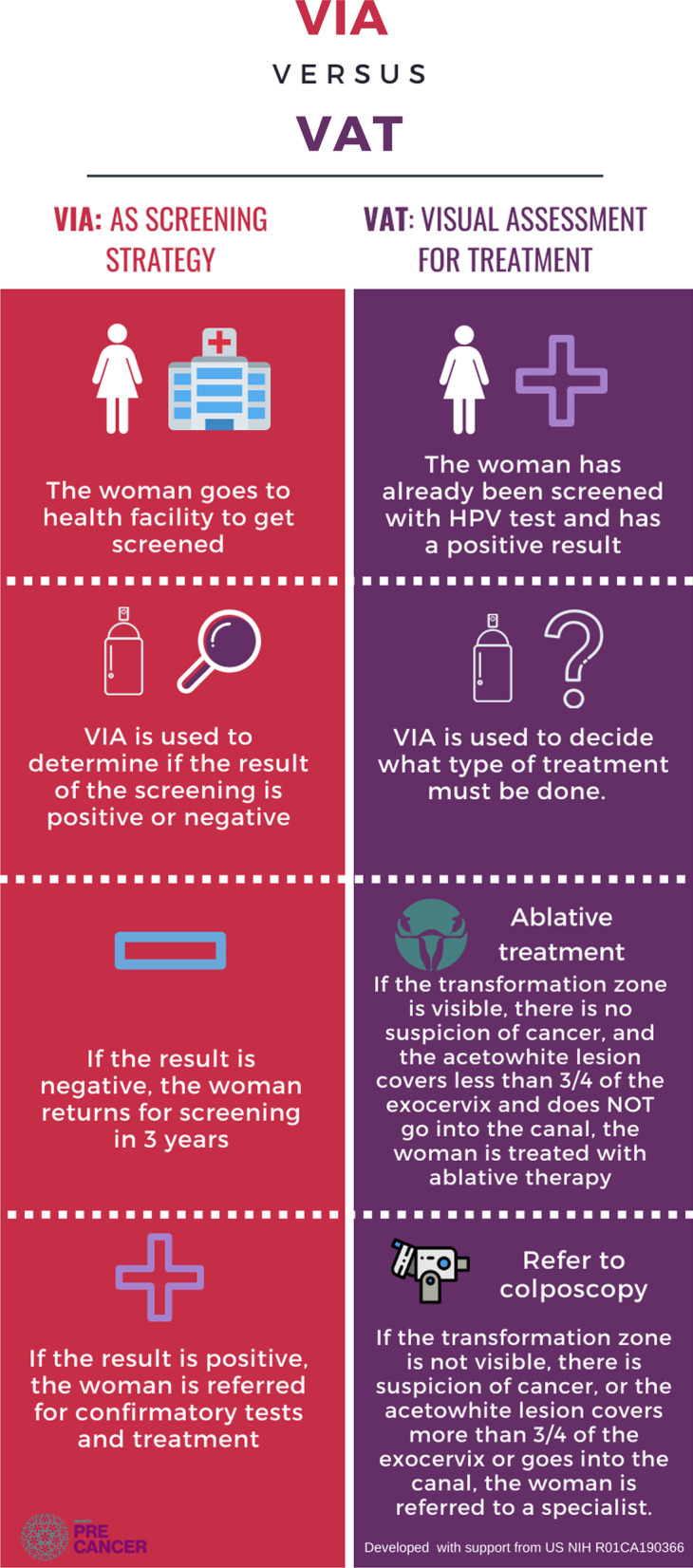
Decision-making process for visual inspection with acetic acid and visual assessment for treatment (i.e., TVT)

Based on these guidelines and suggested technologies, Proyecto Precancer—an implementation science project based in the Micro-Red Iquitos Sur (MRIS) health network in Loreto, Peru—facilitated deliberative dialogues with the regional health authorities and professionals to understand current system challenges in the EDT program. The project used findings from this process to inform the development, planning, and implementation of a new EDT program based on a screen-and-treat approach [14]. This new EDT approach consisted of HPV testing followed by visual triage and ablative therapy treatment for eligible women (e.g., no visible cervical lesions or lesions < 75% of the cervix) or referral to hospital-level specialists for ineligible women (e.g., lesions covering > 75% of the cervix, suspected cancer cases, lack of visibility of the transformation zone of the cervix, or lesions which extend into the endocervical canal) (see Fig. 1). The process of visual triage to determine eligibility for ablative therapy is referred to throughout this paper as the Spanish acronym: TVT-TA where TVT refers to visual triage (*“triaje visual para tratamiento”*) and TA refers to ablative therapy (*“terapia ablativa”*). Ablative therapy was selected as the mode of treatment by health professionals in the MRIS due to portability of the required equipment (it is lightweight, battery operated, and fits in a small handbag). This is particularly important in areas of the MRIS where few roads exist and most transportation is done by river. Cryotherapy was discussed as a possible treatment method; however, it requires transporting bulky, heavy gas containers to remote areas in the MRIS to treat women and shipping containers to and from Lima, Peru to be refilled. This process is costly, time consuming, and cumbersome.

Ablative therapy destroys precancerous tissue by placement of a reusable heated probe on the cervix for 20–40 s [2] and is 93.8% effective at treating cervical cancer intraepithelial neoplasia (CIN)—a precancerous lesion grade 2 or more (CIN2+) [15]. Previous studies have outlined quantitative descriptions of the experience of ablative therapy, with most focusing on the physical side effects and reporting only mild to moderate adverse effects (e.g., vaginal discharge) [15–21]. Only a small number of studies have qualitatively described women’s experiences of ablative therapy and perceptions of the acceptability of ablative therapy; these studies have found that ablative therapy is a feasible and acceptable means of treating HPV according to the women receiving the treatment [20, 21]

Concerns about the potential of overtreating women in screen-and-treat programs have been expressed, primarily by the medical community [22]. However, evidence from LMICs reveals that screening rates can be improved through HPV screening due to the option of self-testing, and follow up and treatment rates can be improved through screen-and-treat options that increase the number of women completing the continuum of care [23, 24]. In support of this, in the MRIS, our monitoring and evaluation data found only 30.2% (52) of VIA screen-positive women received the proper follow up care. Implementation of TVT-TA in the MRIS aimed to address many of the reasons for women not completing the continuum of care by task shifting the management of cervical cancer screening and precancer treatment from the hospital-level to the primary-level health facilities. Following implementation of the TVT-TA program, our monitoring and evaluation data found 70.0% (406) of HPV screen-positive women received the proper follow up care [25].

To further understanding of the factors underlying the successful adoption of the TVT-TA program, this study aims to ensure that the recipients of this type of program—women—have a voice in the process. As such, our study explores the experiences of women with positive HPV results before, during, and after the TVT-TA procedure at the primary-level to determine the acceptability and feasibility of this program in a Peruvian context.

## Methods

This qualitative study took place in the Loreto district (population 1.1 million), located in the Northeast Amazonian region of Peru. Cervical cancer death rates in this region are among the highest in South America [3, 5]. Specifically, the largest health network of the capital city of Iquitos (population 415,000) is the MRIS that covers the San Juan Bautista district (population 127,000), with ~ 20,000 women between the ages of 30–49 years [26]. These women qualify for the screen-and-treat approach with HPV testing and, if eligible, ablative therapy or, if not eligible, referral to follow up care or excisional treatment.

### Participant selection and procedures

**Fig. 2:**
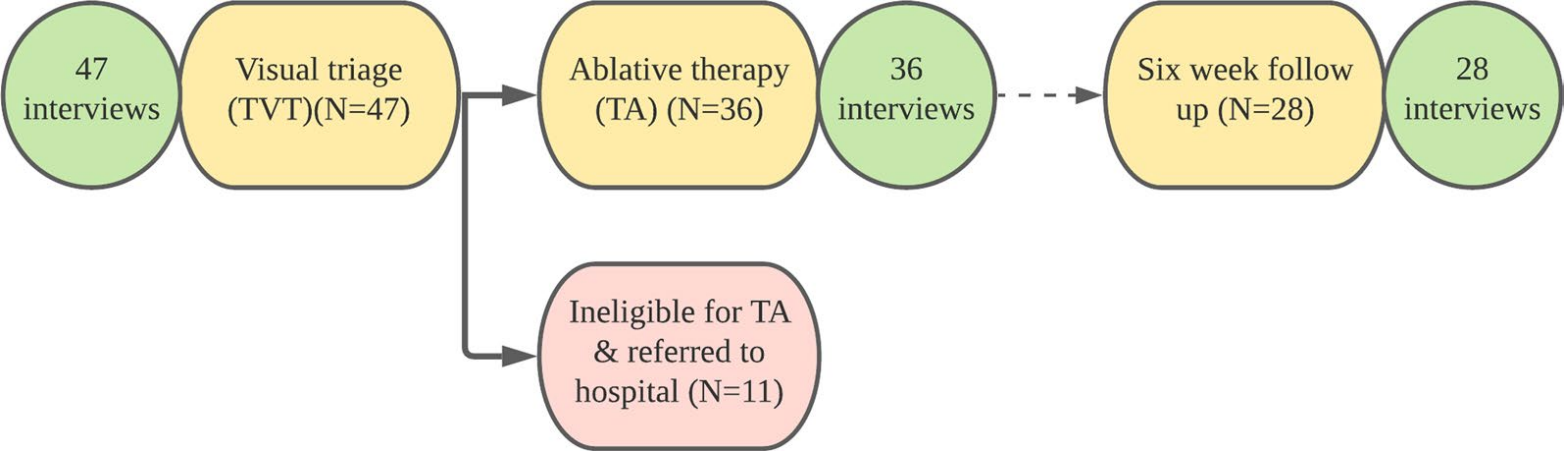
A summary of interviews and the TVT-TA procedure

We purposively recruited women who were eligible for participation between 30 and 49 years old and had a positive HPV screening test (M_age_ = 37.77 years, SD_age_ = 6.49). Women presented at one of the two participating health centers in the MRIS for the TVT-TA procedure within three months of their screening. All women who received TVT were offered the option of follow up with ablative therapy at that moment (if eligible) or to continue their follow up and care at the hospital level. Counseling prior to TVT-TA focused on describing pros and cons to both types of follow up. If women decided to receive ablative therapy at that time, they then signed a consent form (routinely administered by the health facility). All women who were interviewed chose to participate in the TVT-TA procedure. Semi-structured interviews were conducted immediately before, immediately after, and six weeks after women’s TVT-TA procedure. Recruitment stopped once 28 six-week post-TVT-TA interviews had been conducted. A summary of the number of interviews conducted at each stage of the process is outlined in Fig. 2.

During TVT, a healthcare provider applied 0.05% acetic acid to evaluate acetowhite changes and visualized the cervix. During ablative therapy, the Liger Medical Thermocoagulator (model: HTU- 110C) was used. The device was used with 16-mm flat tip or 19-mm nipple tip removable probes, as appropriate. A summary of the TVT-TA procedure including the number of women who received treatment and the number who were referred to the hospital is also summarized in Fig. 2.

### Data collection

We designed a semi-structured interview guide. Questions before the TVT-TA appointment covered participants’ understandings of and feelings surrounding a positive HPV result and the TVT-TA procedure. Immediately following the procedure, questions focused on levels of pain, post-procedure education, and areas for improvement in women’s experiences. The six-week follow up interview investigated side effects of treatment, adherence to post-procedure recommendations, patient education, and finally, whether the participant would recommend the procedure or not and why or why not.

A visual analog pain scale was used to collect quantitative data about pain levels experienced throughout the procedure. Women were shown the scale and asked to rank the pain they experience quantitatively on an 11-point scale from 0 to 10 with corresponding descriptive words (0 being no pain at all and 10 unbearable pain). Women were asked in the interview immediately following TA about their pain during TA and their pain immediately after TA. Women were asked in the six week follow up interview about their pain in the days and weeks following TA.

The interviewer (E.J.R.L.) was a Peruvian research assistant, with a background in nursing, from Loreto with extensive experience in qualitative interviews. Upon initiating the interview, she provided background on Proyecto Precancer and described the objective of the interview. The interviews, conducted in the participants’ native language, Spanish, took place from September 2019 through December 2019 in a private area within the primary care center where TVT-TA took place. After written consent was obtained, these interviews were audio recorded and transcribed verbatim. Field notes were taken and used to inform follow up interviews but not for data analysis.

To ensure protection of privacy and confidentiality of data, once transcribed, all identifiable information was removed from interview transcripts, and the audio files were deleted. De-identified transcripts were stored on password-protected computers. Only the study investigators had direct access to the original transcripts. Quantitative data were collected on paper and uploaded onto secure computers at the study project offices. These offices have long-standing measures in place for minimizing risk of breach of confidentiality of data stored on paper or electronically. The cervical cancer screening registry staff and the research investigators were the only individuals with direct access to the data and results collected.

### Data analysis

Data analysis was an interactive process, completed by two researchers in tandem (S.S., J.B.). The researchers used Dedoose Version 8.0.35 and analyzed the interview transcripts using thematic analysis. They developed a code book by reviewing interviews and highlighting new and existing themes as they emerged. All transcripts were double-coded, and any coding differences were discussed between the coders and resolved. Minor additions and edits were made to the codebook during the coding process. Coders then reviewed all transcripts to ensure they were coded in line with the final version of the codebook. Consolidated criteria for reporting qualitative research were employed to enhance qualitative reporting [27]. Descriptive statistics regarding participants’ pain scores were conducted in R version 4.0.2.

## Results

### Physical experience

The women who received TVT-TA as part of this study reported their physical symptoms qualitatively and quantitatively during, immediately after, and six-weeks after the TVT-TA procedure. The mean amount of pain, on a scale of 0 (none) to 10 (highest), during, immediately after, and six weeks later was 3.53, 1.79, and 1.21, respectively (Table 1).

**Table 1:** The physical experience of pain as reported by women during and after ablative therapy

	During ablative therapy (*N* = *34*^b^)			Immediately after ablative therapy (*N* = *34*^b^)			Six weeks after ablative therapy (*N* = *28*)		
Pain score^a^ 0 (none)–10 (unbearable)	Mean	SD	Range	Mean	SD	Range	Mean	SD	Range
	3.53	2.14	1–10	1.79	0.98	1–6	1.21	0.42	1–2

*N* number, *SD* standard deviation

^a^Although our analog pain score was from 0 to 10 and some women explicitly mentioned in their interviews not having experienced pain during the TVT-TA procedure, no women reported a pain score of 0. See limitations section for possible explanations

^b^Two women who received TVT-TA did not report quantitative pain scores

Women who received TA reported a moderate pain experience (M = 3.53, SD = 2.14) during the 40 s procedure. There was a large range of pain sensation, from very little (1) to unbearable (10). Most women compared the TA experience to menstrual cramps—from light to severe—as well as a burning and pulling sensation:

Like a menstrual cramp. It hasn’t been a strong pain or anything like that. (Participant 2, 33 years old)

There has been little pain. My pain was as if they were pulling something. (Participant 33, 47 years old)

[I would give it] an 8. It would hurt, I would calm down, it was a strong pain… as if I was giving birth. (Participant 41, 33 years old)

Women also commonly reported that they imagined the TA procedure would be much more painful than it was in reality. In fact, several women reported they had been told this procedure would be painful, even when the procedure was new in Iquitos. It is possible that they had been told about a different procedure or were confused about the procedure. One woman described being told that “*they are going to cauterize the tube of the uterus*” (Participant 11, 47 years old) and another described expecting the treatment to be pills:

I was expecting capsules here, pills, “you're not going to do that, you're not going to eat that”; I mean, in my ignorance, I thought a lot of things, but in the end, it wasn't like that. (Participant 7, 35 years old)

Overall, women were pleasantly surprised with the manageable discomfort of the TA procedure:

I have heard what they say about the pain, that I would not tolerate it, but there has been none of that. The obstetra was talking to me while I was there and then left. I practically did not feel anything. (Participant 44, 42 years old)

Yes, I was nervous because…I imagined something else, that it was going to be painful, stronger [pain]. I don’t know, something like that, but no. The treatment was simple. (Participant 9, 34 years old)

Upon exiting the procedure room after TA, women reported a mean pain scale score of 1.79 (SD = 0.78), with a range of very little (1) to moderate (6) pain. However, most women described “*no pain, I feel calm, normal, the way I felt when I came in*” (Participant 26, 31 years old) or mild pain similar to “*the urge to urinate*” (Participant 31, 38 years old).

At the six week follow up, women reported a mean pain scale score of 1.21 (SD = 0.42), with a range of very little (1) to a little (2) pain. Women also reported moderate yet tolerable menstrual cramp-like pain, beginning at post-treatment day two and lasting for a few days. One woman described her pain as “*menstruation pain*” (Participant 24, 30 years old) while other women stated:

What I felt was like a pain, like a menstrual cramp; during the whole day I felt that sensation as if I was going to get my period, I mean, like a cramp… it was a pain that was there, but it was not an exaggerated pain, it was like a mild cramp, a menstrual cramp. (Participant 7, 35 years old)

As if I was going to get my period, not so much I would say, I mean, the pain was mild… it must have been about a week, I mean, it was getting less and less. The pain was moderate, it was bearable. (Participant 18, 47 years old)

Six women reported purchasing pain medications (e.g., ibuprofen) from pharmacies outside of the health centers:

Participant: Yeah, uh-huh, [I took] diclofenac with paracetamol.

Interviewer: And who prescribed that one?

Participant: I bought it. (Participant 21, 46 years old)

Sometimes I felt pain because I took pure ibuprofen and naproxen to stop the pain. (Participant 26, 31 years old)

One woman reported taking natural remedies in addition to pain medication (Participant 19, 33 years old). During the interviews, many women described how they had anticipated the possibility of experiencing side effects, as they had been informed they might experience about four weeks of bleeding during the pre-TVT-TA counseling.

I didn’t call [the obstetra] because they told us that this [bleeding] was normal. And now, if we were to bleed and have a fever, to go immediately to the health center, but as I told you, I have bled, but I have not had a fever. (Participant 1, 49 years old)

I was bleeding for a week. I was like that. The doctor also explained to me that this was normal. (Participant 39, 39 years old)

Because the lady [the obstetra] explained to me all that I'm going to see. After that, I'm going to see bleeding. Everything she has told me, yes, I have seen. (Participant 32, 37 years old)

21 women reported post-TA bleeding; however, five women reported heavy bleeding and were surprised and even scared by the amount of bleeding. The onset and duration of the bleeding varied, ranging from 1-3 days or 1–2 weeks. Most of the women who bled after TVT-TA were not surprised or concerned about the bleeding; however, the five women who experienced heavy bleeding reported going to the health post with concerns about the bleeding:

I have had to use Pampers [diapers] because the Serena [pad] was not enough. I used Serena and it would go through. When I would get up, it [blood] would come down, as if this was leaking… I bled a lot. Sometimes I was afraid to go out in this condition. (Participant 5, 35 years old)

Women also commonly spoke of an unanticipated vaginal odor described as the smell of “*rotten meat*,” “*fish*,” “*bleach*,” “*medicine*,” or “*cheese*.” Six women reported going to the health post prior to their scheduled six-week follow up appointments, and two women reported calling their obstetra for reassurance about their pain level or vaginal discharge. Four of these women were prescribed pain medication.

### Emotional experience

**Fig. 3:**
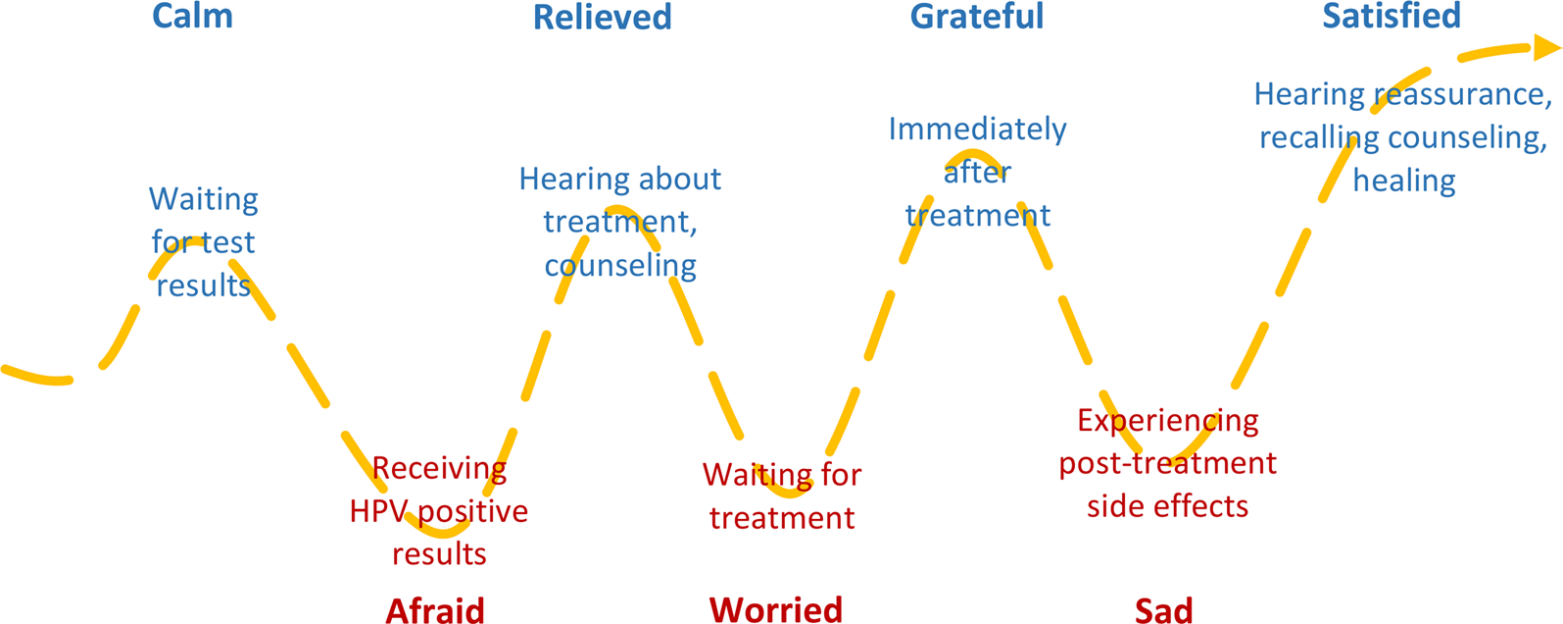
The emotional cycle starting at HPV testing through ablative therapy

This study also probed on the emotional response to the TVT-TA process, including finding out about a positive HPV test, waiting for follow up, and receiving TVT-TA (Fig. 3). Women cycled between fear and relief as they learned they had high risk HPV and then heard that there were potentially simple and fast treatments that could be done outpatient at the primary level.

Regarding their feelings when they heard of their positive test results, all women reported feeling scared, and some reported sadness, crying, and anger.

At the start I felt a bit bad, I felt sad. I didn’t know if I should cry or be angry. (Participant 7, 35 years old)

It’s like a bucket of water. It’s something I don't know how to explain, sudden news. I got a little depressed. I felt bad. (Participant 44, 42 years old)

I felt tense, half scared, how am I going to have that? I was worried. I couldn’t believe I had it. I was worried. (Participant 45, 33 years old)

Though a positive HPV result does not indicate cancer, women commonly associated positive HPV results with cancer, and four women remembered a loved one who died from cervical cancer.

I received [the positive test results] poorly. I felt very bad, just with the word “cancer” I feel terror, because my mother died of that, of uterine cancer. They told me I have the virus, that if I don’t do treatment, from here to 10 years cancer could develop. Psychologically to me this is something terrible. (Participant 21, 42 years old)

"Don’t cry," the obstetra said to me. But who can think that? Because I have a cousin that died; it has not even been a month since we buried her ... she died with cancer, only a matter of time. (Participant 11, 47 years old)

Prior to treatment, some women felt extremely nervous. They worried about their positive HPV results and any pain associated with the procedure.

Because I imagined that they were going to hurt me, that suddenly it was going to be a pain that I couldn’t stand, that I was going to cry… It felt like with the Pap, only of course that it has been a little bit painful, nothing more. I mean, it was something I did not expect, I imagined something worse. (Participant 7, 35 years old)

Most women appreciated counseling by the obstetra, which decreased nervousness and fear by teaching about the virus and what to expect in treatment.

With the obstetra’s explanation, she explained more clearly, in detail, then I calmed down because she told me that it's not bad yet. (Participant 10, 41 years old)

They have made me understand that it is not the disease, but the virus, that I am on time. (Participant 26, 31 years old)

I was a bit surprised, but when she was explaining it to me, I calmed down because I thought that, as they say, cancer, that it was already cancer and she told me that no, it was just the virus, but that's what the treatment is for, and that's why she referred me here. (Participant 18, 47 years old)

Other women, however, did not understand or were too scared to focus on the information provided in the obstetra counseling. When asked about whether counseling about the treatment was provided, one woman responded:

Yes, I think she explained to me. Because I was a little bit scared, I think I forgot. (Participant 29, 46 years old)

After the procedure, all women expressed relief and happiness knowing that they could eliminate their HPV, decreasing their chances for future cervical cancer. They appreciated what they described as good treatment, with no surprises, by caring obstetras.

It was as I imagined because they had already given me a talk previously [during the delivery of results]. (Participant 24, 30 years old)

I say better because now I know that I have the treatment. (Participant 9, 34 years old)

I feel better, because they are giving me a good result that will be for my good. (Participant 22, 33 years old)

### Reflections on the process

The most common sentiment described regarding the treatment process was that women were pleased with the process, with many women commenting on relief at how quickly their positive test results were managed, and others commenting on the relief at not having to live with the anxiety of waiting for future tests and treatments.

They always look at me and ask me if it hurts. "No," I tell them, "it hasn't hurt me.” I have withstood everything they have done to me. Yes, I feel at ease now because I am already undergoing treatment. (Participant 32, 37 years old)

I felt comfortable, and it was better because I felt like when I get the treatment I am going to be healed. (Participant 27, 50 years old)

The quick resolution offered by TVT-TA was commonly mentioned in the interviews. Sixteen women explicitly said they were pleased with the time between screening and treatment.

But, as soon as the results came back, the doctors did not even take a week to do this for us… Them [health providers] knowing that the results were positive, they have rushed to do this [treatment] on time and quickly. We have had one month and a half and already, already we are healing… Thanks to God that they have treated us fast and we are being treated and already healing… (Participant 11, 47 years old)

The attention, right away. When I had the treatment, I will tell you that I felt relieved. (Participant 44, 42 years old)

I see it as a good thing because it is only a few days, two days. I have only had to wait two days for my treatment to begin. (Participant 41, 33 years old)

A few of the women spoke about the decrease in the amount of time needed for the entire screening to treatment process in comparison to their previous experiences with other forms of cervical cancer screening.

Faster than a test... a Pap smear… takes a month to two months [between screening and results]. (Participant 20, 37 years old)

I think it has been good, fast, because when I did the Pap smear, they made me delayed, how long I have waited, after a year that... I got the results, after a year. (Participant 11, 47 years old)

Almost all women mentioned that they would recommend the procedure to other women.

“Well,” I tell them [neighbors who found out that I had this disease], “I was scared, but I recommend this,” I tell them. “It is good to know what it is and even much better when they tell you that you will have treatment that instant.” I thought a lot and got scared, I even lost my appetite… But thinking about it well… I prefer that they give me a treatment instead of worrying about having this disease… As I said, I am not embarrassed to tell them [neighbors], yes, I tell them, I tell them what it is. What my treatment has been. Thank God it has all been done in one instant…” (Participant 32, 37 years old)

I have already recommended it to several people, as I said, to my aunt, to my cousin, to my sisters. I have also told them that it is good. (Participant 2, 33 years old)

Yes, I have told several of them, my neighbors, when they came to see me, I have told them "why don't you have it done? Do your treatment on time so you know what you have... "Yes, well" they have told me, "but... but that treatment that you have had in particular, it costs, almost 400 [soles]" a neighbor told me... I tell them that, "send them to have it done, right now, it doesn't cost anything, everything is free... That is what I tell neighbors... I tell them, "Do it on time, follow your treatments. (Participant 11, 47 years old)

Many of the women also expressed a desire for the TVT-TA strategy to continue and be expanded so that treatment can reach more women.

Let them continue with this because many women in Loreto are getting sick. (Participant 18, 47 years old)

[I would say] that this test be extended in the region. Because it cannot be my case alone, but of how many women within our city of Loreto, that may be like me. Suddenly, they do not know because we may seem like we are fine on the outside... Well for me, my advice would be to expand this method and this treatment for many women who do not know about this virus that I could have in my body. But suddenly there are cases of women who are more advanced and who do not know about it or suddenly out of fear, fear, they do not go to a health center. (Participant 7, 35 years old)

Overall, there was a tremendous amount of positive feedback and gratitude for the obstetras’ and doctors’ hard work and the TVT-TA method.

Thank you because there is this method that can save us; well, not all of us have the same luck, others are already too advanced in their disease because some are afraid of being checked or touched, but no one lives in fear, on the contrary, we must try to take care of ourselves more, to be able to control ourselves more as women on the outside and inside, let's say, on the inside. (Participant 26, 31 years old)

To the doctors who are linked to this program, I ... tell them that there are many people who have benefited a lot through this program and that this should not be lost, that it should not disappear. There are many people, like me, humble people who have nothing and through this program they are able to benefit, to have these treatments and to be healed. (Participant 44, 42 years old)

## Discussion

Most women in this study reported moderate pain during TA and minimal pain immediately after TVT-TA and six weeks after. Pain scores reported during TA were commonly lower than the women themselves had expected, and most compared the pain experience to that of menstrual cramps. The most frequent physical complaints post treatment were bleeding and vaginal odor associated with TA. Women reported oscillating emotions starting from receiving their positive HPV results through treatment and healing after TVT-TA: from fear associated with receiving their positive HPV results, relief upon hearing about TVT-TA as a treatment, worry about the TVT-TA procedure itself, calmness with counseling, and relief after the treatment. The post treatment relief was strongly emphasized by participants who were pleased with the treatment despite any experienced pain or side effects. Nearly all participants mentioned that TVT-TA was easier and faster than expected and would recommend that TVT-TA continue to be expanded to more women throughout the region.

TA has been found to be an acceptable, safe, and feasible form of treatment in a variety of settings, including LMICs [15]. The majority of research on TA has not considered the adverse effects of treatment; however, the studies that have reported adverse effects have rarely found serious adverse effects associated with treatment with the majority of studies reporting only mild to moderate adverse effects (e.g., vaginal discharge) [15–21]. When considering adverse effects during TA, previous studies have reported pain during TA with women reporting moderate pain (10.5%) and severe pain (3.5%) [15]. Additionally, one study reported the following adverse effects during TA: cramps (79% of women), heat sensation in the vagina (25% of women), mild bleeding (2% of women) and vasovagal reaction (2% of women) [18]. A further study corroborated that mild cramping was the most frequent adverse event during ablative therapy [20]. Our study similarly found pain, cramps, and a heat sensation to be common experiences during TA.

When considering adverse effects following TA, a previous study specifically examined adverse effects at 1-month post-TA and found that nearly all women reported watery, vaginal discharge for 1–3 weeks and that a minority of women reported bleeding [16]. An additional study found the following adverse effects at 1-month post-TA: mild pain (4.2% of women), mild bleeding (21.9% of women; mean duration 3 days), and vaginal discharge (25.0% of women; mean duration 10 days) [19]. In contrast to these studies, our study found that a majority rather than a minority of women reported post-TA bleeding; however, our study corroborates the findings regarding vaginal discharge post-TA. It is possible that discrepancies between this study’s findings and the previous literature result from a limited body of previous literature where the post-TA adverse effects have not been substantially demonstrated. Further research is needed to substantiate the findings regarding adverse effects of TA.

Our original pre- and post-TVT-TA counseling materials did not include specific reference to the potential for prolonged vaginal bleeding and odor. Based on the results of this study, we revised the counseling materials to include these potential side effects. We found that the level of concern regarding the post-treatment bleeding and odor was reduced when pre-treatment counseling included these expectations. However, further research is needed to understand and systematically document adverse effects associated with TVT-TA and to establish ways in which these adverse effects can be safely addressed. We recommend that future studies evaluate these adverse effects and consider ways in which this information can be included in counseling for women pre-TA.

Most studies evaluating TA as a method for treating HPV include a confirmatory biopsy [15]. Our study, however, does not include further confirmatory screening, largely due to resource and time constraints in the MRIS. Our monitoring and evaluation of the MRIS system found that only 30.2% of women seeking follow up care following a positive VIA screening result before our project began completed the continuum of care, commonly due to barriers including unavailability of providers, broken equipment, out of pocket payment for medicine, and cost related to travel or missing work. Implementation of the TVT-TA program allowed task shifting the management of cervical cancer screening and precancer treatment from the hospital-level to the primary health facilities removing barriers associated with the complicated and time-consuming hospital-level system and increasing the rate of women who completed the continuum of care to 70.0%. Thus, while critics cite overtreatment as a downside to HPV screen-and-treat programs [22], in resource limited contexts, we provide further evidence that HPV screening followed by TVT-TA is a viable early detection and treatment method. Further, this study provides evidence that, despite the potential of overtreatment as a result of TVT-TA, risk associated with this procedure is outweighed by the relief provided by having completed the procedure; women mentioned very few negative, long-term side effects and consistently recommend TVT-TA continue and be expanded to more women throughout the region.

### Limitations

In qualitative studies, the presence of the researcher can affect the response of participants, the collected data can be interpreted differently by researchers, and the number of interviews relies on a limited sample size. In order to minimize these effects, the interviews were conducted by a local obstetra who has spent many years working in the healthcare system. Data were coded and analyzed by two researchers and compared to account for any discrepancies, and the interviews led to comprehensive themes that were repeated by the women, indicating saturation. It is possible that translations from Spanish to English may have changed the meaning in the results reported. However, analysis was conducted in Spanish, and only the final results were translated into English by a bilingual English/Spanish speaker. These data were gathered from a specific context and the exact effects of TVT-TA may not be generalizable to a broader population. The data do, however, provide valuable information about the general acceptability and feasibility of TVT-TA. Lastly, women in this study were presented with a visual analog pain scale and asked to rank the pain they experienced quantitatively on an 11-point scale from 0 (no pain) to 10 (unbearable pain). Notably, no women in this study reported a score of 0 (no pain); however, some women explicitly mentioned during interviews that they experienced no pain during part of the TVT-TA procedure. It is therefore possible that some women may have interpreted the scale as a 10-point scale from 1 (no pain) to 10 (unbearable pain); if so, this bias would only mean that the physical pain experience was even lower on the scale than what we report. Additional quantitative research is needed to clarify exact pain responses to TVT-TA. Despite this, whether 1 or 0 indicates no pain, this study provides a general indication of the pain levels experienced during TVT-TA.

## Conclusion

This paper explored the feasibility and acceptability of TVT-TA among those most affected: women who test positive for HPV in a resource-limited setting. We found the TVT-TA program to be a safe and acceptable means of preventing future development of cervical cancer. We recommend that future research further explore the adverse effects associated with ablative therapy to ensure comprehensive pre-procedure counseling. Further, this research supports the WHO’s updated guidelines on TA treatment [2] and can continue to inform international guidelines on treatment for patients with HPV in resource-limited settings.

## Data Availability

Data and materials are available on request to the corresponding author.

## Abbreviations

TVT-TA: *Triaje visual para tratamiento y terapia ablativa* [Visual triage for treatment and ablative therapy]
LMIC: Low- and middle-income country
WHO: World Health Organization
HPV: Human papillomavirus
EDT: Early Detection and Treatment
MRIS: Micro-Red Iquitos Sur
CIN: Cervical cancer intraepithelial neoplasia
